# Deep phenotyping of socio-emotional skills in children with typical development, neurodevelopmental disorders, and mental health conditions: Evidence from the PEERS

**DOI:** 10.1371/journal.pone.0291929

**Published:** 2023-10-11

**Authors:** Vicki Anderson, Simone Darling, Stephen Hearps, David Darby, Julian Dooley, Skye McDonald, Lyn Turkstra, Amy Brown, Mardee Greenham, Louise Crossley, George Charalambous, Miriam H. Beauchamp

**Affiliations:** 1 Murdoch Children’s Research Institute, Parkville, Victoria, Australia; 2 Royal Children’s Hospital, Parkville, Victoria, Australia; 3 University of Melbourne, Parkville, Victoria, Australia; 4 Florey Institute of Neurosciences, Royal Parade, Parkville, Victoria, Australia; 5 PSI School, Twinsburg, Ohio, United States of America; 6 University of New South Wales, Sydney, New South Wales, Australia; 7 School of Rehabilitation Science, McMaster University, Hamilton, Ontario, Canada; 8 Curve Technology, Royal Children’s Hospital, Parkville, Victoria, Australia; 9 University of Montreal, Montreal, QC, Canada; 10 Sainte-Justine Hospital Research Center, Montreal, QC, Canada; University of Montenegro, MONTENEGRO

## Abstract

**Objective:**

Socio-emotional skills, including social competence and social cognition, form the basis for robust relationships and wellbeing. Despite their importance, these skills are poorly defined and measured, particularly in children with developmental vulnerabilities. As a consequence, targets for effective management and treatment remain unclear. We aimed to i) phenotype social competence and social cognition in typically developing children (TDC) and in children with neurodevelopmental or mental health disorders (clinical groups) and ii) establish the relationships between these child-direct measures and parent ratings of social competence and behavior.

**Method:**

Using a multi-site, cross-sectional study design, we recruited 513 TDC and 136 children with neurodevelopmental (autism spectrum disorder [ASD], attention deficit hyperactivity disorder [ADHD]) or mental health (Anxiety Disorder [ANX]) diagnoses (age range 5–15 years). We administered the Paediatric Evaluation of Emotions, Relationships and Socialisation (PEERS) to children, and parents completed standardised questionnaires rating children’s socio-emotional function.

**Results:**

Standardised parent questionnaires revealed a global pattern of everyday socio-emotional impairment that was common to all clinical groups, while PEERS identified disorder-specific socio-cognitive profiles for children with ASD, ADHD and ANX. Compared to TDCs, children with ASD demonstrated global socio-cognitive impairment. Children with ADHD were impulsive, demonstrating difficulties managing speed accuracy trade-offs. Children with ANX exhibited slowed social decision-making, but otherwise intact skills.

**Conclusions:**

Standardized parent questionnaires of child socio-emotional function reveal differences between children with typical and atypical development, but do not yield disorder-specific, socio-emotional profiles. In contrast, findings from the PEERS objective assessment suggest that that ASD, ADHD and ANX are associated with distinct socio-cognitive phenotypes, to more accurately guide and target management and treatment of impaired social competence.

## Introduction

Socio-emotional skill is an umbrella term encompassing both ***social competence*,** the behaviors needed to develop satisfying, lasting relationships, and ***social cognition*,** reading each other’s faces, actions, and gestures to determine what others are thinking and feeling.

Underpinning the development of these skills is a complex interplay of a distributed neural ‘social network’, cumulative social and environmental exposures and multiple neurocognitive processes and resources [1–5]. With increasing focus on well-being, and the importance of early identification of socio-emotional vulnerabilities it is imperative to accurately identify and understand both normal and impaired social competence and social cognition [6, 7] to accurately guide management and treatment.

### Social competence

Social competence has been defined as the level of ability a child possesses to successfully coordinate and implement the processes and resources available to meet social demands and achieve social goals (e.g., peer interactions) [8–10]. Environmental factors, and family influences in particular (e.g., parents’ attitudes, beliefs, social role models, parent-child interactions), contribute significantly to the development of social competence through childhood [11]. With a handful of recent exceptions, social competence is assessed using parent- or teacher-rated, ‘broad-band’ questionnaires [12] (e.g., Social Skills Improvement System [SSIS] [13]; Child Behavior Checklist [14]; Strengths and Difficulties Questionnaire [SDQ] [15], School Social Behavior Scale (SSBS-2) [16] or diagnosis-specific tools (e.g., Autism Spectrum Rating Scales [17]). These measures provide insight into everyday function, but not to the socio-cognitive skills that underpin social competence.

### Social cognition

Social cognition refers to “those aspects of higher cognitive function which underlie smooth social interactions by understanding and processing interpersonal cues and planning appropriate [social] responses.” [18. p.559]. These processes underpin the ability to perceive and process social information and include recognizing others’ thoughts and emotions, attributing mental states to oneself and others, understanding that others have mental states different from one’s own and attributing causes or intent to others’ behaviors [11]. Social cognition skills emerge gradually through infancy into adolescence [19], beginning with infant smile and imitations of others’ actions [20]. The ability to recognize and understand emotions and theory of mind emerge later, around three to four years [21–23] and develops through childhood. Complex social information processing, social communication and intent attribution mature later and, by the end of adolescence, most young people are capable of high-level social decision making [24, 25].

Targeted assessment of social cognition, using tools that precisely characterize the child’s ‘socio-cognitive profile’ is critical for precise, effective intervention. With a few exceptions (e.g., NEPSY Affect Recognition and Theory of Mind [26]), tools evaluating children’s social cognition have inadequate normative data, are designed primarily for adults and have limited appeal for children (e.g., Ekman faces [27]; Mind in the Eyes Test, [28]; Trolley Task, [29]). Optimally, tools assessing children’s social cognition should take account of developmental factors and incorporate real-life situations, viewed from a first-person perspective, to maximize ecological validity [7, 12, 30].

### Disruptions to socio-emotional development

Maturation of social competence and social cognition is dependent upon a healthy environment, strong social models, and a healthy brain [11]. Disruptions to development, due to biological or environmental factors, increase the risk of derailing these skills, though the magnitude of the impact may be difficult to accurately quantify due to imprecise definition and assessment [1, 3, 4].

To date, most research has focussed on social competence, employing either dimensional approaches such as observer ratings, which place the child’s social competence along a continuum, or diagnostic tools (e.g., Diagnostic Statistical Manual of Mental Disorders–V [31], which classify a child’s function in terms of symptom clusters. Based on such approaches, global estimates suggest 50–60% of children with neurodevelopmental disorders, (NDDs) [32, 33] and 10% of children in the general population experience impaired social competence [34]. Elevated risk of impaired social competence is also reported in children with acquired brain injury [35–39], epilepsy [40], chronic illness [41], learning disabilities [42], attention-deficit/hyperactivity disorder (ADHD) [43], and anxiety [44]. Consequences of deficits in social competence include social isolation and exclusion, bullying, lack of satisfying friendships, behavior problems and poor quality of life.

In contrast, apart from ASD research, little attention has been paid to the social cognitive mechanisms underpinning impaired social competence or to whether specific socio- cognitive ‘phenotypes’ can be identified for NDDs and child mental health conditions which can then be used to guide, targeted, effective treatments to optimize socio-emotional development. Further, the question remains as to whether these problems represent a primary social cognitive impairment or a secondary consequence of other conditions and comorbidities.

Our study objective was to comprehensively assess and characterize social competence and social cognition in typically developing children (TDC) and children with neurodevelopmental and mental health (‘clinical’) diagnoses (ASD, ADHD, anxiety [ANX]) using novel tools able to distinguish diagnosis-specific socio-emotional profiles. Specifically, we predicted that: i) compared to TDC, children with clinical diagnoses would be differ on both parent ratings and child-direct assessment; ii) standardised parent questionnaires would not differentiate between the clinical groups and instead yield a global, non-specific profile of socio-emotional impairment common to all groups; and iii) child-direct assessment would yield evidence for distinct, disorder-specific “*social cognition profiles*,*” w*hich could represent novel and potentially modifiable targets for personalised treatment in these high-risk populations.

## Materials and methods

### Design

This study employed a multi-site, cross-sectional design and recruited a community-based sample of TDC, and a clinical sample of children with NDDs and mental health diagnoses. Participants in the TDC sample and clinical groups are mutually exclusive.

### Participants

#### Community-based TDC (n = 513)

Data were collected at public, private, and Catholic primary and high schools (2016–17) across the state of Victoria, Australia. Schools were approached based on student enrolments (>100, <1500), location (metropolitan, regional), and socioeconomic status (SES, using the Index of Community Socio-Educational Advantage [45]) to provide a range of demographic backgrounds. Inclusion criteria included: (a) children aged 5–15 years, (b) enrolled in mainstream school, and (c) competent in English. Parents or children with insufficient English to complete the study requirements were excluded. Medical and developmental histories were documented based on parent report. Those with neurodevelopmental or mental health diagnoses confirmed by a psychologist, psychiatrist, or pediatrician were then included in the clinical sample group.

#### Clinical sample (n = 136)

Children with ASD (*n* = 53), ADHD (*n* = 50) and ANX (*n* = 33) were identified from the study community-based sample, and from referrals to outpatient Behaviour Problems and Neurodevelopmental Clinics at The Royal Children’s Hospital (RCH), Melbourne. Inclusion criteria for the clinical sample were: (a) children aged 5–15 years and; (b) have a formal diagnosis, based on DSM-V criteria (i.e., from pediatrician, psychiatrist, psychologist) of ASD, ADHD or ANX. Parents or children with insufficient English to complete the study requirements were excluded. Families of eligible children identified via outpatient clinic lists were approached at a clinic visit and provided with study information. Consenting families had an appointment scheduled to complete the child’s assessment.

### Measures

#### Sample characteristics

*Clinical history and demographics*. Parent/primary caregivers completed a questionnaire detailing child medical, developmental and educational history, gender (which was collapsed into male/female sex due to small numbers of other genders recorded), race and mental health status. The Index of Relative Socio-economic Disadvantage (IRSD) [46] was completed as a measure of SES, with higher scores indicating greater social disadvantage.

*Intellectual function (IQ)*. A Full-Scale IQ was derived through administration of the age-appropriate version of the Wechsler scales: 5 years, Wechsler Preschool and Primary Scale of Intelligence-Fourth Edition general ability index (GAI: Information, Similarities, Block Design, Matrix Reasoning) (WPPSI-IV) [47]; ≥6 years, Wechsler Abbreviated Scale of Intelligence- Second Edition two-subtest full scale intelligence quotient (FSIQ-2: Vocabulary, Matrix Reasoning) (WASI-II) [48].

*Child behavior*. The Strengths and Difficulties Questionnaire: parent form (SDQ) [15] measures parent perception of the child’s emotional and behavioral status (4–17 years), providing Total Difficulties, Internalizing and Externalizing behaviors scores and five subscales (Emotional Symptoms, Conduct Problems, Inattention/Hyperactivity, Peer Problems, and Prosocial Behavior). Scores were categorized as follows: normal: within 1 standard deviation (SD) of the TDC group mean; borderline-clinical problems = 1–2 SD above the TDC mean; clinical problems: > 2 SD above the TDC mean.

#### Social competence

*Social Skills Improvement System, parent form (SSIS) [13].* is a widely-used, broad-band measure of children’s social skills (3–18 years) [12]. The Social Skills domain was employed in this study (*M* = 100, *SD* = 15) to determine the convergent validity of PEERS and PEERS-Q measures.

*PEERS-Questionnaire*. Parent (PEERS-Q) [49, 50] assesses everyday social competence, with a focus on real life socio-cognitive skills. It comprises a total score and six subscales: Relationships, Participation, Social Rules, Social Communication, Social Cognition and Behaviour (*M* = 50, *SD* = 10), with scores >65 indicating ‘clinical’ problems.

#### Social cognition

**Fig 1:**
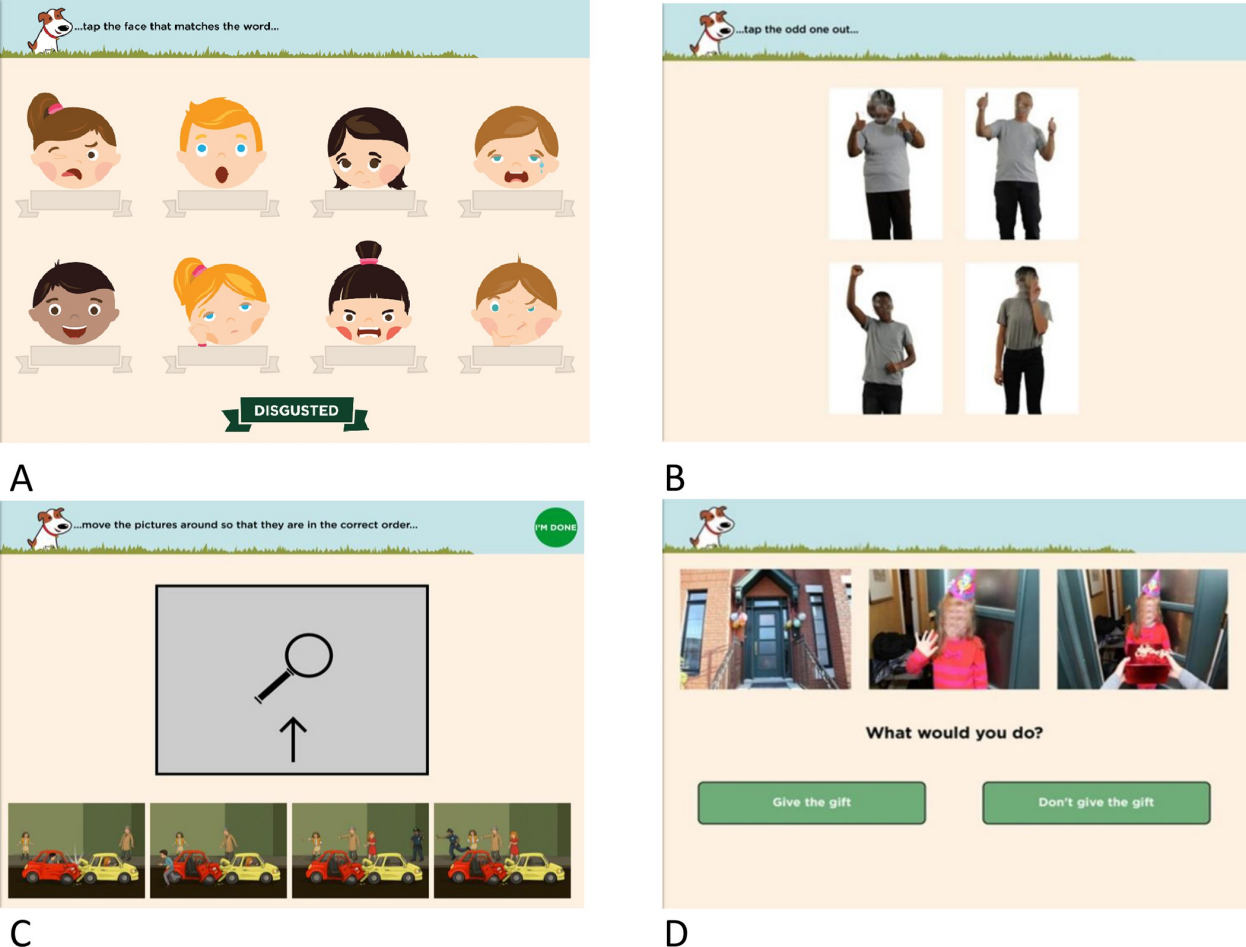
Examples from the PEERS subscales: A. Matching Emo, B: Odd One, C. Get This, D. Multiple Morals.

*Paediatric Evaluation of Emotions Relationships and Socialisation (PEERS)*. PEERS [51, 52] is a child-direct, app-based, interactive digital health tool delivered via iPad providing a sex and age-normed assessment of social cognition, social communication and attention/executive skills, using real-life situations, viewed from a first-person perspective, to maximize ecological validity. PEERS includes three sex and age-adjusted composite scales (Cognition, Primary Social Processing, Complex Social Processing, *M* = 100, *SD* = 15), a Total score (*M* = 100, *SD* = 15) and nine subtests (*M* = 10, *SD* = 3) and three Supplementary subtests which do not contribute to the Total Score. (Table 1, Fig 1). Each subtest begins with a practice item, followed by the scored test. PEERS software automatically calculates sex and age-normed scores based on error and completion times scores from the Community-based TDC sample (referred to as the ‘normative sample’).

**Table 1:** Pediatric evaluation of emotions, relationships and socialization.

SUBSCALE	SUBTEST	SKILL	LEVEL	DESCRIPTION	VARIABLES
**COGNITION**	Friend Find	Selective attention	Primary	Selective and divided attention	Errors; Completion time
	Move Fast	Reaction time	Primary	Response speed	Completion time
	Think Fast	Processing speed	Primary	Processing speed for socio-emotional labels	Errors; Completion time
	Matching Emo	Emotion perception	Primary	Knowledge/recognition of emotional labels	Errors; Completion time, Linear Integrated Speed-Accuracy Score (LISAS)
**PRIMARY SOCIAL PROCESSING**	Odd One	Emotion recognition- gestures	Primary	Recognition of non-verbal gestures	Errors; Completion time, LISAS
	Social Scenes	Social perception	Primary	Interpretation of social intent	Errors; Completion time, LISAS
	Finding Emo	Emotion recognition–faces	Primary	Facial emotion recognition	Errors; Completion time, LISAS
**COMPLEX SOCIAL PROCESSING**	Get This	Social interpretation	Complex	Understanding social cues	Errors; Completion time Get This score
	Multiple Morals	Moral Reasoning Theory of mind	Complex	Moral decision-making and reasoning in social situations	Decision Making; Moral Maturity; Emotional Congruence
SUPPLEMENTARY TESTS *(Children older than 8 years only)*	Say What	Prosody	Complex	Understanding social cues in conversation	LISAS
	Mind Read	Theory of Mind	Complex	Physical and mental state Theory of mind	Levels 1,2,3: Errors, Completion time, Mind Read Score
	Social Intent	Social information processing	Complex	Intent attribution Response bias	Hostile; Non-Hostile; Aggressive; Passive; Positive; Negative, Social Information Processing score

#### Cognitive composite scale

*Friend find (selective attention)*. Two trials of 40 photos, headshots depicting various genders, ages and ethnicities, from multiple angles, showing different emotions, with a ‘target photo’ at the top of screen. In each trial, 12 photos are of the target. The participant selects targets as quickly as possible. Errors and completion time are recorded. Omission and commission errors are summed to measure total error count.

*Move fast (reaction time)*. One trial comprising 40 stimuli (e.g., balls), appearing consecutively on the screen at random locations. The participant taps each stimulus as quickly as possible. Reaction times for each response are summed to measure completion time.

*Think fast (processing speed)*. One trial of 40 illustrated (cartoon) faces (5x8 grid) displaying an emotion (happy, sad, angry) appear on the screen in random order. The participant taps the face and names the emotion out loud, starting from top left, and left to right. The assessor records incorrectly labelled emotions, and sums for a total error count. Completion time is also recorded.

*Matching emo (emotion perception)*. 12 trials (Fig 1A), each consisting of eight illustrated (emoji) faces (4x2 grid), displaying one of 12 emotions, with the target emotion labelled at the bottom of the screen. The participant selects the face matching the target emotion by dragging the label to the correct face. Errors and completion time are recorded and used to generate a Linear Integrated Speed-Accuracy Score (LISAS).

#### Primary social processing composite scale

Errors and completion time are recorded and used to generate a LISAS for all subtests.

*Odd one (social intent in nonverbal gestures)*. 11 trials, with four photos (full body, 2x2 grid) (Fig 1B). Three photos display the same emotion, and one a different emotion. The participant selects the discordant emotion.

*Social Scenes (social actions and intentions)*. Nine trials consisting of four photos (2x2 grid), three displaying a scenario with the same social theme (e.g., bullying), and one a different social theme. The participant selects the discordant theme.

*Finding emo (emotion recognition)*. 12 trials of four photos of faces (2x2 grid). Three photos display the same emotion, and one shows a different emotion. The participant selects the photo displaying the discordant emotion.

#### Complex social processing composite scale

*Get this (social interpretation)*. Eight trials of four illustrated (cartoon) pictures, with participants asked to order the pictures to make sense of the social scenario (Fig 1C). Order, errors and completion times are recorded. The ‘Get This’ score is calculated based on the ordering of the pictures (correct order = 6 points; pictures 1 and 4 ordered correctly = 2 points; pictures 2 and 3 are correct = 1 point).

*Multiple morals (socio-moral reasoning, theory of mind, modified from Beauchamp et al. [24]).* (Fig 1D). The child views three static photographs depicting an everyday socio-moral dilemma and is asked: “What would you do?”, with “Do/Don’t do” options relevant to the scenario (Decision Making score); followed by “Why?”, with a choice of six responses (Moral Maturity score, based on [53]) and “How would you feel?” with a choice of 10 emotions displayed as cartoons (Emotional Congruence score). Correct responses are summed to generate the three summary scores.

#### Supplementary measures

Three supplementary subtests assess complex socio-cognitive skills (prosody, theory of mind, intent attribution) and were administered to children eight years and older.

*Say what (prosody recognition, modified from the awareness of social inference test, [54]).* 14 trials, participants listen to a short audio recording of a content-neutral sentence spoken with an emotional tone, then identify the tone from a list of seven emotions. Errors, time to completion and LISAS are calculated.

*Mind read (theory of mind, adapted from Turkstra et al. [55]).* Three conditions: 1) single video vignettes showing actors in social interactions (four trials); 2) paired video vignettes showing similar interactions, with the answer to the second vignette in each pair depending on correctly answering the first (four trials); and 3) paired vignettes, but with videos in each pair separated by a 30-second distracter (five trials). Participants watch each vignette and answer a yes/no question displayed on the screen (e.g., “Does he mean what he said?”). Errors and response times are recorded for each condition, and a ‘Mind Reading’ score is generated by summing the errors from the first question in each pair from conditions 2 and 3.

*Social intent (complex social processing, intent attribution, adapted from Dooley et al. [56]).* The participant watches six short videos of social interactions with ambiguous intent. For each, four multiple choice questions are displayed on the screen, regarding whether the interaction was hostile or non-hostile (question 1), passive, aggressive or assertive (question 2 and 3), and positive or negative (question 4). Hostile, aggressive and negative responses are summed to produce a “Social information Processing score”, with higher scores representing poorer social information processing.

### Procedure

This study was approved by the Human Research Ethics Committees of the Victorian Department of Education and Early Childhood (002318), Catholic Education Melbourne (2166) and The RCH (34046), which conform to the Declaration of Helsinki. The individuals depicted in Fig 1 of this manuscript have given written informed consent to publish these images. Schools selected to provide a demographically representative sample, were approached and provided with a study overview. Those agreeing to participate distributed study information and consent forms to parents of all enrolled children (electronic/ hardcopy). Consenting parents returned forms either to the school in hardcopy or electronically via a secure research database (REDCap) [57]. If questionnaires were not received on the day of the assessment, parents were sent daily reminders for four days. If questionnaires were still outstanding, the research team made two reminder phone calls, after which the data were considered missing.

Once parents returned consent forms, an assessment was scheduled either at the child’s school, or outpatient clinic at The RCH. Children 12 years and older provided written consent, and children younger than 12 years provided verbal assent. Assessments were conducted individually by trained researchers beginning with the IQ assessment followed by PEERS and took on average 1–1.25 hours. Parent ratings and child-direct assessment data were available for all children in the sample.

### Statistical analysis

Analyses were conducted using Stata v15.1 [58]. Means and standard deviations were generated for continuous variables, frequencies and percentages for categorical variables. Planned contrasts of clinical groups compared to the TDC group were conducted, with independent *t*-tests comparing continuous measures, and chi-squared tests for categorical data.

Group means for the TDC and Clinical groups were compared for intellectual ability, social cognition, social competence alongside 95% confidence intervals (95% CI), allowing comparison of measurement and accuracy of clinical group scores to normed values. Planned contrasts between TDC and each clinical group were explored using independent *t*-tests. For these analyses, a value of *p*<0.05 was deemed statistically significant.

## Results

### Sample characteristics

Participant demographics, FSIQ data, and parent-ratings are provided in Tables 2 and 3. For the TDC sample, mean age was 8.9 years (*SD* = 2.4), 357 (69.6%) were of Caucasian origin, 79 (15.4%) Asian origin, with the remainder including small numbers of African, Indigenous, and Hispanic children. In the clinical sample, 116 (85.3%) of children were of Caucasian background, with 8 (5.9%) of Asian heritage, 4 (2.9%) were Indigenous, 1 (0.7%) African descent, with race unspecified for 7 (5.1%). SES was comparable across groups, with the exception that the ADHD group recorded lower SES (*p* = 0.006). For FSIQ, all group means fell within the average range, but for ASD and ADHD groups, mean FSIQs were lower than the TDC (*p*s <0.001).

**Table 2:** Sample demographics.

	TDC	ASD	ADHD	ANXIETY
N	513	53	50	33
Sex (female), *n* (%)	263 (51.3)	**12 (22.6)** *	**13 (26.0)** *	16 (48.5)
Age (years), *M (SD*)	8.9 (2.4)	**10.3 (3.0)** *	**10.2 (2.6)** *	**11.6 (3.1)** *
Ethnicity, *n* (%)				
Caucasian	357 (69.6)	**44 (83.0)** *	40 (80.0)	**32 (97.0)** *
Indigenous Status	79 (15.5)	5 (9.4)	2 (4.0)	1 (3.0)
Asian	3 (0.6)	0 (0.0)	4 (8.0)	0 (0.0)
African	8 (1.6)	1 (1.9)	0 (0.0)	0 (0.0)
Hispanic	8 (1.6)	0 (0.0)	0 (0.0)	0 (0.0)
Other	43 (8.4)	3 (5.7)	4 (8.0)	0 (0.0)
SES (IRSD), *M (SD)*	1019.7 (44.0)	1009.6 (51.7)	**996.8 (56.5)** *	1023.3 (44.4)

TDC = typically developing children, ASD = autism spectrum disorder, ADHD = attention deficit/hyperactivity disorder, SES = socioeconomic status, IRSAD = Index of Relative Socio-economic Advantage and Disadvantage, M = mean, SD = standard deviation.

* *p*< 0.05; compared to TDC

Note: TDC group is mutually exclusive from clinical groups.

**Table 3:** Comparison of intellectual ability, behavior and social competence across TDC and clinical groups.

	TDC			ASD			ADHD			ANXIETY		
	N	M	(95%CI)	N	M	(95%CI)	N	M	(95%CI)	N	M	(95%CI)
Full Scale IQ*	513	103.2	(102.1–104.2)	31	92.0	(85.6–98.3)	39	90.2	(85.6–94.8)	15	101.1	(90.2–111.9)
**SDQ**												
Total	451	7.0	(6.5–7.5)	29	21.4	(19.0–23.7)	32	21.0	(18.4–23.6)	17	20.7	(17.2–24.2)
Emotional symptoms	451	1.9	(1.7–2.1)	29	5.4	(4.5–6.4)	32	4.5	(3.5–5.5)	17	7.0	(5.7–8.3)
Peer problems	451	1.0	(0.9–1.2)	29	3.5	(2.4–4.6)	32	4.3	(3.3–5.3)	17	2.5	(1.4–3.5)
Conduct problems	451	2.9	(2.6–3.1)	29	7.5	(6.7–8.4)	32	8.1	(7.4–8.9)	17	6.8	(5.3–8.3)
Hyperactivity/inattention	451	1.2	(1.1–1.4)	29	4.9	(4.2–5.6)	32	4.1	(3.2–4.9)	17	4.5	(3.6–5.3)
Prosocial	451	8.6	(8.4–8.7)	29	6.3	(5.4–7.2)	32	6.3	(5.6–7.1)	17	7.2	(5.9–8.5)
Internalising	451	3.1	(2.9–3.4)	29	10.4	(9.0–11.8)	32	8.5	(6.9–10.2)	17	11.5	(9.7–13.3)
Externalising	451	3.9	(3.6–4.2)	29	11.0	(9.3–12.7)	32	12.5	(11.0–14.0)	17	9.2	(7.1–11.4)
**SSIS**												
Social Skills	452	102.5	(101.2–103.9)	32	75.8	(70.0–81.7)	39	77.4	(72.3–82.4)	19	80.9	(72.0–89.8)
**PEERS-Q**												
Total	459	49.3	(48.3–50.2)	30	72.1	(68.7–75.4)	33	71.4	(68.0–74.8)	17	66.7	(60.5–72.9)
Relationships	459	49.8	(48.8–50.7)	30	69.5	(66.6–72.4)	33	67.2	(63.6–70.8)	17	65.7	(60.5–70.9)
Participation	459	49.4	(48.5–50.4)	30	66.4	(63.2–69.7)	33	62.4	(59.3–65.5)	17	65.0	(59.6–70.3)
Social Rules	459	49.5	(48.6–50.5)	30	67.1	(62.6–71.7)	33	69.3	(65.6–73.0)	17	59.0	(51.9–66.2)
Social Communication	459	49.2	(48.2–50.1)	30	66.7	(63.8–69.5)	33	65.6	(62.2–68.9)	17	63.3	(57.0–69.6)
Social Cognition	459	49.2	(48.2–50.2)	30	68.3	(64.9–71.7)	33	67.8	(64.7–70.9)	17	62.3	(56.1–68.5)
Behaviour	459	49.3	(48.3–50.2)	30	67.8	(63.8–71.7)	33	69.0	(65.4–72.6)	17	63.8	(56.2–71.3)

TDC = typically developing children, ASD = autism spectrum disorder, ADHD = attention deficit/hyperactivity disorder, SDQ = Strengths & Difficulties Questionnaire, SSIS = Social Skills Improvement System, PEERS-Q, Paediatric Evaluation of Emotions, Relationships and Socialisation Questionnaire, M = mean, 95%CI = 95% confidence interval.

*Only Full Scale IQ for the anxiety group was not significantly different to TDC (*p* = 0.21).

For clinical groups, SDQ Total Difficulties scores were similar, and in the ‘clinically significant’ range for all clinical groups (ASD: *M* = 21.4; ADHD: *M* = 21.0; ANX: *M* = 20.7) (see Table 3). In keeping with expectations, on the SDQ, children with ASD and ANX were rated as having internalizing behaviors in the clinical range, with borderline-clinical externalizing symptoms. In contrast, the ADHD group had clinical level externalizing behaviors and borderline-clinical internalizing symptoms. Prosocial and peer problems were only detected in the ASD and ADHD groups. Of note, all clinical group mean ratings fell in the clinical range for the hyperactive/ inattentive subscale, and only the ANX group had mean scores in the clinical range for Emotional Symptoms.

### Social competence: Parent ratings

On *broad band social competence questionnaires* (SSIS: Social skills, PEERS-Q), parents endorsed significantly more problems across all clinical groups compared to TDC (all *t*-test comparison *p*s<0.001) (See Table 3). Mean parent ratings on the SSIS Social Skills (ASD = 75.8, ADHD = 77.4, ANX = 80.9) fell in the borderline-clinical range for all clinical groups. In contrast, PEERS-Q provided a more fine-grained description of everyday social competence. PEERS-Q total scores were in the clinically significant range for ASD (*M* = 72.1) and ADHD (*M* = 71.4), and borderline-clinical for ANX (*M* = 66.7). In addition, parents’ endorsements of children with ASD were in the clinical range on all subscales. ADHD group ratings were similar, with the exception that Participation scores were borderline-clinical. The ANX group’s PEERS-Q subscale scores were less elevated, falling in the borderline-clinical range, apart from normal ratings for Social Rules, and clinical level ratings for Relationships.

### Social cognition: Child-direct assessment

Age-standardised PEERS Total, Composite and subtest scores are detailed in Table 4, and raw scores are presented in S1 Table.

**Table 4:** Comparisons of PEERS performances between TDC and clinical groups–scaled and composite scores.

	TDC			ASD			ADHD			ANXIETY		
	N	M	(95%CI)	N	M	(95%CI)	N	M	(95%CI)	N	M	(95%CI)
***Cognition***												
Friend Find	513	9.6	(9.3–9.9)	**53**	**8.4**	**(7.5–9.3)** *	**50**	**8.4**	**(7.5–9.2)** *	33	9.6	(8.6–10.6)
Move Fast	513	9.5	(9.2–9.7)	53	9.2	(8.3–10.1)	**50**	**8.6**	**(7.8–9.4)** *	33	9.3	(8.2–10.3)
Think Fast	513	9.5	(9.3–9.8)	**53**	**8.4**	**(7.6–9.2)** *	50	8.8	(7.9–9.7)	33	8.9	(7.9–10.0)
Matching Emo	513	9.6	(9.4–9.9)	**53**	**8.6**	**(7.8–9.3)** *	50	9.0	(8.1–9.8)	33	8.7	(7.6–9.8)
*TOTAL COMPOSITE SCORE *	513	96.4	(95.1–97.8)	**53**	**89.2**	**(84.7–93.6)** *	**50**	**89.5**	**(85.1–94.0)** *	33	93.1	(87.4–98.7)
***Primary Social Processing***												
Odd One	513	9.1	(8.8–9.3)	**53**	**7.3**	**(6.4–8.2)** *	**50**	**6.8**	**(5.8–7.9)** *	**33**	**7.9**	**(6.4–9.4)** *
Social Scenes	513	9.2	(9.0–9.5)	**53**	**8.0**	**(7.2–8.9)** *	**50**	**7.3**	**(6.4–8.2)** *	33	9.1	(8.0–10.1)
Finding Emo	513	8.9	(8.7–9.2)	**53**	**7.6**	**(6.7–8.5)** *	**50**	**7.2**	**(6.2–8.1)** *	**33**	**7.1**	**(5.9–8.2)** *
*TOTAL COMPOSITE SCORE *	513	95.0	(93.8–96.3)	**53**	**86.2**	**(82.4–90.0)** *	**50**	**83.5**	**(79.4–87.5)** *	**33**	**88.6**	**(83.3–94.0)** *
***Complex Social Processing***												
Multiple Morals	278	9.6	(9.2–10.0)	38	8.7	(7.6–9.7)	39	9.1	(8.0–10.1)	28	9.0	(8.0–10.1)
Get This	57	9.4	(8.5–10.2)	26	10.5	(8.9–12.2)	29	8.8	(7.5–10.0)	18	10.3	(8.7–12.0)
*TOTAL COMPOSITE SCORE *	278	96.3	(94.8–97.7)	39	96.3	(91.4–101.2)	40	94.1	(88.9–99.2)	28	97.2	(92.1–102.3)
*PEERS TOTAL SCORE*	513	94.3	(93.0–95.6)	**53**	**83.7**	**(78.6–88.9)** *	**50**	**81.3**	**(75.7–86.9)** *	**33**	**87.8**	**(81.7–94.0)** *
***Supplementary Tests***												
Say What	57	10.0	(9.2–10.8)	25	8.7	(7.4–10.0)	**29**	**7.4**	**(6.3–8.6)** *	18	9.8	(8.2–11.5)
Mind Read	38	9.6	(8.6–10.6)	15	9.9	(8.2–11.7)	20	8.8	(7.4–10.2)	12	10.6	(8.8–12.3)
Social Intent	40	10.4	(9.5–11.4)	**22**	**7.9**	**(6.8–9.0)** *	**26**	**7.1**	**(6.0–8.2)** *	**15**	**8.2**	**(6.6–9.8)** *

TDC = typically developing children, ASD = autism spectrum disorder, ADHD = attention deficit/hyperactivity disorder, M = mean, 95%CI = 95% confidence interval. Note: TDC group is mutually exclusive from clinical groups.

*Bold indicates significant group differences, *p*< .05. **Not applicable

#### PEERS total and composite scale scores

Significant differences were found between the TDC sample and the clinical groups across the PEERS Total and the three Composite Scale mean scores. For PEERS Total, ASD and ADHD group means were more than 10 points (that is, 2/3 SD) below the TDC group and significantly lower on all PEERS Composite scales (all *t*-test comparison *p*s<0.001) apart from the Primary Social Processing Composite. In contrast, the ANX group recorded similar results to the TDC group for PEERS Cognitive and Complex Processing Composites, with only the PEERS Total and Primary Social Processing Composite deviating significantly from TDC.

#### PEERS subscale scores

Comparison of *Cognitive Composite subtest* means found the ASD group achieved lower results than the TDC for Friend Find (*p* = 0.006), Think Fast (*p* = 0.011), and Matching Emo (*p* = 0.01), while the ADHD group had lower scaled scores for Friend Find (*p* = 0.006) and Move Fast (*p* = 0.039). In contrast, the ANX group recorded faster completion times than the TDC group for all *t*-tests (all *p*s≤ 0.023), except for Matching Emo. Examination of subtest raw scores (S1 Table) identified no meaningful group differences for mean errors.

On the *Primary Social Processing Composite Scale*, all mean subtest scores were below the TDC for the ASD (all *p≤*0.015) and ADHD groups (all *p<*0.001). The ANX group demonstrated lower results than TDC for Odd One (*p* = 0.027) and Finding Emo (*p<* 0.001). There were no differences in raw scores between ASD and TDC groups (all *p*>0.07). The ADHD group made more errors than TDC on Odd One and Finding Emo (*p* <0.05), and faster completion times on Finding Emo (*p* = 0.006) and Social Scenes (*p* = 0.032), suggesting a speed accuracy trade-off.

Results of the *Complex Social Processing Composite Scale* subtests results varied. There were no significant differences between ANX and TDC groups, but, on the Composite score, ASD and ADHD subgroups once again scored significantly poorer than TDC (*p*<0.01). On Multiple Morals the ASD group recorded significantly lower scores for Moral Maturity and Emotional Congruence (*p* = 0.024 and *p*<0.001, respectively). Raw scores identified longer completion times and lower overall scores on the social interpretation subtest Get This for children with ASD, and more errors and lower overall scores for the ADHD group. The ANX group recorded slower raw scores for completion times on Get This compared to TDC.

### Supplementary subtests

For *Say What*, only the ADHD group performed significantly worse than TDC (*p<*0.01) overall, with performances characterized by more errors and slower completion times. The ASD group were less proficient in recognizing emotions in conversation. LISAS scores, integrating speed and accuracy, were significantly lower than TDC for all clinical groups. For *Mind Read*, scaled scores were similar across all four groups, and raw scores found no differences between TDC and ANX groups except for completion time for Level 2 (10.5 seconds, *p* = 0.022). The ASD group had slower completion times than TDC at all three conditions of the task, making more errors at Condition 1. Children with ADHD made more errors at Condition 1 and 3 and were slower on Condition 2. All clinical groups performed worse than TDC on *Social Intent*, with the ADHD group recording the greatest differences, followed by the ASD group (both *p*<0.001). Raw scores indicated more hostile, aggressive and negative (all *p*<0.01) responses than TDC for both ADHD and ASD groups. For the ANX group, subtest scores were lower than TDC, but only the Social Information Processing Score result reached significance (*p*<0.001).

## Discussion

This study explored whether a novel digital assessment battery social cognition could differentiate specific socio-cognitive profiles across TDC and children with ASD, ADHD and ANX to facilitate more accurate understanding of the bases of everyday problems in social competence and thus contribute to targeted management and treatment for deficits in social competence. As predicted, comprehensive, child direct assessment identified distinct ‘socio- cognitive profiles’ reflecting underlying characteristics of impaired social cognition and characterized by differences in nature and level of social cognitive abilities. In keeping with the generic nature of parent-ratings [12, 56, 59], we expected that, compared to TDC, parents of children with clinical diagnoses would report global, clinically significant problems in their child’s everyday social competence. Our findings partially supported these predictions, with parent endorsements sensitive to the presence of reduced social competence, but not sufficiently precise to differentiate specific social competence patterns for clinical groups. In contrast, parents were able to identify specific *behavior profiles* for internalizing (ASD, ANX) and externalizing behaviors, in keeping with their child’s diagnosis.

### Parent ratings of social competence and behavior

Parent ratings of children’s *social competence* distinguished clinical and TDC groups. Ratings of social skills (on SSIS) [13], while elevated, were not in the clinical range. In contrast, on PEERS-Q, which provides a fine-grained description of social competence [49], total scores were in the clinical range for all clinical groups. Further, for ASD and ADHD groups, clinical-level problems were present across all subscales, other than borderline-clinical participation for the ADHD group. For the ANX group, ratings were generally borderline-clinical with clinical problems for Relationships and normal ratings for Social Rules.

### Social cognitive skills

Child direct assessment provided additional detail regarding social cognitive abilities and was able to distinguish social profiles across TDC and clinical groups. Consistent with expectations and parent ratings, *the ASD profile* was characterized by significant challenges in primary social processing, including recognizing, processing and labelling emotions across modalities (verbal, gesture, visual), as well as processing and interpreting simple social scenes. For complex social processing, moral maturity, emotional congruence, theory of mind, intent attribution and social information processing were all impaired compared to normative data (S1 Table). These poor results were exacerbated by cognitive difficulties, notably slowed information processing.

The *ADHD profile* included reduced cognition and primary social processing underpinned by specific weaknesses in cognitive efficiency and self-regulation, rather than primary social cognitive deficits. Children with ADHD recorded relatively faster response times and more errors when identifying emotions and interpreting social aspects of real-life situations, suggesting they struggled to manage speed-accuracy trade-off effectively (S1 Table). On complex intent attribution tasks, children with ADHD were more likely to interpret others’ actions as hostile or negative, rather than positive or neutral. Such responses may reflect prior negative social experiences, with ADHD associated with elevated risk of bullying by peers [32].

*Children with ANX* demonstrated relatively few difficulties in social cognition, in contrast to NDD groups and consistent with previous research [60]. Primary social processing difficulties were mild and specific to emotional labels, including facial and gestural representation of emotions. Consistent with the ADHD group, an intent attribution bias was identified, with this group also more likely to interpret others’ actions as hostile or negative, rather than positive or neutral. This is in line with findings that identified that those with social anxiety have a tendency to focus more on negative emotions in comparison to positive emotions [61]. Together, these findings suggest weaknesses, or hesitancies, in interpreting basic emotions, but intact abilities for complex social decision-making.

These distinct, disorder-specific social profiles provide meaningful information, superior to that of parent ratings, regarding the nature of social problems across clinical groups. This more nuanced characterization contributes to our understanding of whether impaired social competence is a ‘primary’ deficit, as for the ASD group, or reflects secondary consequences, as with ADHD, where social problems were characterized by reduced response inhibition and impulsivity and difficulties balancing the speed-accuracy trade-offs. Our findings are similar those from adult research that suggests performance on social cognitive tasks varies more subtly in those with ADHD compared to ASD [62]. In contrast to ADHD and ASD, and despite reports of day-to-day social difficulties, children with ANX had overall intact social competence, apart from slowed response times on some social information processing tasks, raising the possibility that social problems in these children may be secondary to anxiety and associated lack of confidence or inefficient decision making. Better understanding of the bases of everyday social behaviors will facilitate the implementation of intervention which best targets primary impairments.

Despite including a large sample and multiple measurement approaches, this study had some limitations. By excluding children attending special needs schools, it is likely that children with more severe ASD, ADHD and anxiety were not recruited. Thus, our findings likely reflect a relatively high-functioning subset of these children, thus restricting our ability to identify diagnosis-specific social profiles for more severe forms of these conditions. We were limited by the reliance on clinical diagnoses of ANX, ASD, ADHD, and a lack of dimensional data regarding children’s symptoms, and were unable to explore relationships between social profiles and diagnostic subtypes.

## Conclusions

There is growing recognition of the importance of socio-emotional skills, yet most health professionals and educators rely on parent- or teacher-rated questionnaires to detect social problems, failing to consider the child’s perspective. We found that, although parent questionnaires revealed a global pattern of socio-emotional impairment that was common to all clinical groups, these instruments do not appear to yield disorder-specific social profiles. In contrast, objective child assessment yielded disorder-specific socio-cognitive profiles; suggesting that while ASD is associated with a global, primary deficit in social cognition, socio-cognitive difficulties in children with ADHD and ANX are likely secondary to their primary diagnosis. These preliminary findings suggest that objective PEERS assessment may yield a more comprehensive and nuanced understanding of disorder-specific social-cognitive impairments, which may represent viable targets for treatment of social dysfunction in these high-risk clinical populations.

## Supporting information

S1 Table Comparisons of PEERS performances between normative and clinical samples–raw scores. (DOCX)
