# Supplementary material for: Deep phenotyping of socio-emotional skills in children with typical development, neurodevelopmental disorders, and mental health conditions: Evidence from the PEERS
Source: PLoS One. 2023 Oct 11;18(10):e0291929. doi: 10.1371/journal.pone.0291929 (PMC10566677; doi:10.1371/journal.pone.0291929)
Supplement: S1 Table — (DOCX) [file pone.0291929.s001.docx]

**Supplementary Table 1: Comparisons of PEERS performances between normative and clinical samples – raw scores.**

|  | TDC | | | ASD | | | ADHD | | | ANXIETY | | |
| --- | --- | --- | --- | --- | --- | --- | --- | --- | --- | --- | --- | --- |
|  | N | M | (95%CI) | N | M | (95%CI) | N | M | (95%CI) | N | M | (95%CI) |
| ***Cognition (COG)*** |  |  |  |  |  |  |  |  |  |  |  |  |
| Friend Find |  |  |  |  |  |  |  |  |  |  |  |  |
| Errors | 513 | 0.9 | (0.8-1.1) | 53 | 1.4 | (0.7-2.1) | 50 | 0.9 | (0.4-1.4) | 33 | 0.5 | (0.3-0.8) |
| Completion Time (seconds) | 513 | 52.2 | (50.4-54.0) | 53 | 53.5 | (47.4-59.7) | 50 | 53.3 | (46.8-59.8) | **33** | **43.7** | **(37.0-50.5)*** |
| Move Fast |  |  |  |  |  |  |  |  |  |  |  |  |
| Completion Time | 513 | 24.5 | (24.2-24.9) | 53 | 23.5 | (22.5-24.5) | 50 | 23.5 | (22.6-24.5) | **33** | **22.3** | **(21.3-23.3)*** |
| Think Fast |  |  |  |  |  |  |  |  |  |  |  |  |
| Errors | 513 | 0.8 | (0.7-0.9) | 53 | 0.8 | (0.5-1.2) | 50 | 0.7 | (0.4-1.1) | 33 | 0.5 | (0.2-0.9) |
| Completion Time (seconds) | 513 | 39.3 | (38.1-40.5) | 53 | 38.1 | (34.9-41.3) | 50 | 36.6 | (33.5-39.7) | **33** | **33.0** | **(30.0-36.1)*** |
| Matching Emo |  |  |  |  |  |  |  |  |  |  |  |  |
| Errors | 513 | 1.6 | (1.4-1.7) | 53 | 1.8 | (1.3-2.3) | 50 | 1.5 | (1.1-2.0) | 33 | 1.3 | (0.9-1.7) |
| Completion Time (seconds) | 513 | 70.3 | (67.6-73.0) | 53 | 70.8 | (64.2-77.4) | 50 | 69.5 | (62.8-76.2) | 33 | 64.1 | (55.5-72.7) |
| LISAS | 513 | 5.2 | (5.0-5.4) | 53 | 5.5 | (4.9-6.0) | 50 | 5.5 | (5.0-6.1) | 33 | 5.0 | (4.3-5.7) |
| ***Primary Social Processing (PSP)*** |  |  |  |  |  |  |  |  |  |  |  |  |
| Odd One |  |  |  |  |  |  |  |  |  |  |  |  |
| Errors | 513 | 2.2 | (2.1-2.4) | 53 | 2.8 | (2.1-3.5) | **50** | **2.9** | **(2.2-3.5)*** | 33 | 1.8 | (1.1-2.6) |
| Completion Time (seconds) | 513 | 73.7 | (70.6-76.9) | 53 | 77.8 | (60.1-95.5) | 50 | 69.0 | (55.4-82.6) | 33 | 73.5 | (58.5-88.4) |
| LISAS | 513 | 8.3 | (7.8-8.8) | 53 | 8.6 | (6.4-10.9) | 50 | 7.2 | (5.7-8.8) | 33 | 7.8 | (6.0-9.7) |
| Social Scenes |  |  |  |  |  |  |  |  |  |  |  |  |
| Errors | 513 | 2.9 | (2.7-3.1) | 53 | 3.2 | (2.6-3.8) | 50 | 3.5 | (2.9-4.2) | **33** | **2.0** | **(1.4-2.5)*** |
| Completion Time (seconds) | 513 | 100.1 | (95.8-104.3) | 53 | 107.1 | (87.0-127.2) | **50** | **84.7** | **(74.4-95.1)*** | 33 | 107.6 | (81.9-133.3) |
| LISAS | 513 | 13.9 | (13.2-14.6) | 53 | 14.8 | (11.0-18.7) | 50 | 11.8 | (10.2-13.3) | 33 | 14.9 | (9.5-20.3) |
| Finding Emo |  |  |  |  |  |  |  |  |  |  |  |  |
| Errors | 513 | 2.5 | (2.3-2.6) | 53 | 2.9 | (2.2-3.6) | **50** | **3.1** | **(2.3-3.8)*** | 33 | 2.5 | (1.9-3.1) |
| Completion Time (seconds) | 513 | 73.0 | (70.6-75.4) | 53 | 75.4 | (63.7-87.1) | **50** | **61.8** | **(55.4-68.2)*** | 33 | 64.8 | (55.1-74.5) |
| LISAS | 513 | 7.1 | (6.8-7.5) | 52 | 6.9 | (5.6-8.2) | **49** | **5.8** | **(5.1-6.5)*** | 33 | 6.1 | (5.2-7.0) |
| ***Complex Social Processing (CSP)*** |  |  |  |  |  |  |  |  |  |  |  |  |
| Multiple Morals |  |  |  |  |  |  |  |  |  |  |  |  |
| Decision-Making Score | 278 | 7.0 | (6.8-7.1) | 38 | 6.6 | (5.9-7.2) | 39 | 6.7 | (6.1-7.2) | 28 | 7.1 | (6.5-7.7) |
| Moral Maturity Score | 278 | 27.7 | (27.1-28.3) | **38** | **25.8** | **(23.8-27.7)*** | 39 | 26.6 | (24.6-28.5) | 28 | 26.8 | (24.8-28.7) |
| Emotional Congruence Score | 278 | 3.4 | (3.3-3.4) | **38** | **3.0** | **(2.9-3.2)*** | 39 | 3.2 | (3.0-3.4) | 28 | 3.3 | (3.1-3.6) |
| Get This |  |  |  |  |  |  |  |  |  |  |  |  |
| Errors | 57 | 2.0 | (1.7-2.4) | 26 | 2.2 | (1.4-2.9) | **29** | **2.9** | **(2.2-3.5)*** | 18 | 1.9 | (1.1-2.8) |
| Completion Time (seconds) | 57 | 168.9 | (154.7-183.2) | **26** | **198.4** | **(173.5-223.2)*** | 29 | 190.6 | (176.3-204.9) | **18** | **199.6** | **(167.6-231.6)*** |
| Get This score | 57 | 24.2 | (21.7-26.6) | **26** | **28.8** | **(25.0-32.6)*** | **29** | **29.2** | **(26.2-32.1)*** | 18 | 27.8 | (23.7-31.8) |
| ***Supplementary subtests*** |  |  |  |  |  |  |  |  |  |  |  |  |
| Say What |  |  |  |  |  |  |  |  |  |  |  |  |
| Errors | 57 | 5.5 | (5.0-6.0) | **25** | **6.6** | **(5.7-7.6)*** | **29** | **7.6** | **(6.8-8.5)*** | 18 | 5.7 | (4.6-6.7) |
| Completion Time (seconds) | 57 | 89.6 | (85.4-93.8) | 25 | 96.9 | (85.3-108.4) | **29** | **99.9** | **(89.9-109.9)*** | 18 | 99.5 | (84.4-114.5) |
| LISAS | 57 | 7.3 | (6.8-7.8) | **25** | **9.7** | **(7.0-12.4)*** | **29** | **9.4** | **(7.2-11.6)*** | **18** | **9.4** | **(6.0-12.8)*** |
| Mind Read |  |  |  |  |  |  |  |  |  |  |  |  |
| Level 1 – Mental and Physical Inferences |  |  |  |  |  |  |  |  |  |  |  |  |
| Errors | 40 | 0.5 | (0.3-0.7) | **16** | **1.1** | **(0.6-1.6)*** | **21** | **1.2** | **(0.8-1.6)*** | 13 | 0.5 | (0.2-0.9) |
| Completion Time (seconds) | 40 | 71.7 | (66.8-76.5) | **16** | **81.4** | **(75.3-87.4)*** | **21** | **80.6** | **(74.0-87.2)*** | 13 | 81.2 | (70.5-91.8) |
| Level 2 – Predict without Distractors |  |  |  |  |  |  |  |  |  |  |  |  |
| Errors | 40 | 2.5 | (2.1-2.9) | 16 | 2.3 | (1.5-3.1) | 21 | 2.9 | (2.2-3.5) | 13 | 2.2 | (1.3-3.2) |
| Completion Time (seconds) | 40 | 107.8 | (103.9-111.7) | **16** | **123.5** | **(113.5-133.4)*** | **21** | **117.9** | **(106.5-129.3)*** | **13** | **118.3** | **(107.4-129.1)*** |
| Level 3 – Predict with Distractions |  |  |  |  |  |  |  |  |  |  |  |  |
| Errors | 38 | 1.7 | (1.3-2.1) | 15 | 2.4 | (1.5-3.3) | **20** | **2.5** | **(1.8-3.2)*** | 12 | 1.6 | (1.0-2.2) |
| Completion Time (seconds) | 38 | 141.3 | (134.9-147.8) | **15** | **157.1** | **(143.1-171.1)*** | 20 | 147.0 | (132.5-161.5) | 12 | 150.4 | (139.1-161.8) |
| Mind Reading Score | 38 | 4.2 | (3.6-4.8) | 15 | 4.9 | (3.5-6.2) | **20** | **5.5** | **(4.5-6.5)*** | 12 | 4.0 | (2.8-5.2) |
| Social Intent |  |  |  |  |  |  |  |  |  |  |  |  |
| Hostile | 40 | 2.5 | (2.0-3.0) | 22 | 3.1 | (2.5-3.8) | **26** | **3.9** | **(3.3-4.5)*** | 15 | 3.1 | (2.7-3.5) |
| Non-hostile | 40 | 3.5 | (3.0-4.0) | 22 | 2.8 | (2.1-3.4) | **26** | **2.0** | **(1.4-2.6)*** | 15 | 2.9 | (2.5-3.3) |
| Passive | 40 | 4.7 | (3.9-5.5) | 22 | 4.5 | (3.5-5.5) | 26 | 4.0 | (3.1-4.9) | 15 | 4.4 | (3.0-5.8) |
| Aggressive | 40 | 0.5 | (0.2-0.8) | **22** | **1.6** | **(0.7-2.5)*** | **26** | **2.1** | **(1.0-3.1)*** | 15 | 1.2 | (0.1-2.3) |
| Assertive | 40 | 6.8 | (6.1-7.5) | 22 | 5.8 | (4.6-7.0) | 26 | 5.7 | (4.5-6.9) | 15 | 6.4 | (4.8-8.0) |
| Positive | 40 | 5.7 | (5.5-5.9) | **22** | **4.8** | **(4.2-5.3)*** | **26** | **4.6** | **(4.1-5.2)*** | 15 | 5.4 | (4.9-5.9) |
| Negative | 40 | 0.3 | (0.1-0.5) | **22** | **1.1** | **(0.6-1.7)*** | **26** | **1.3** | **(0.8-1.9)*** | 15 | 0.6 | (0.1-1.1) |
| Social Information Processing Score | 40 | 3.3 | (2.6-4.0) | **22** | **5.9** | **(4.4-7.3)*** | **26** | **7.3** | **(5.7-9.0)*** | **15** | **4.9** | **(3.3-6.5)*** |

TDC = typically developing children, ASD = autism spectrum disorder, ADHD = attention deficit/hyperactivity disorder, M = mean, 95%CI = 95% confidence interval, LISAS = Linear Integrated Speed-Accuracy Score, Social Information Processing Score = sum of all Hostile, Aggressive and Negative responses.

Note: TDC group is mutually exclusive from clinical groups.

*Bold = significant group differences
